# Convolutional Neural Networks for Direct Inference of Pharmacokinetic Parameters: Application to Stroke Dynamic Contrast-Enhanced MRI

**DOI:** 10.3389/fneur.2018.01147

**Published:** 2019-01-08

**Authors:** Cagdas Ulas, Dhritiman Das, Michael J. Thrippleton, Maria del C. Valdés Hernández, Paul A. Armitage, Stephen D. Makin, Joanna M. Wardlaw, Bjoern H. Menze

**Affiliations:** ^1^Department of Computer Science, Technische Universität München, Munich, Germany; ^2^GE Global Research, Munich, Germany; ^3^Department of Neuroimaging Sciences, Centre for Clinical Brain Sciences, University of Edinburgh, Edinburgh, United Kingdom; ^4^Department of Infection, Immunity and Cardiovascular Disease, University of Sheffield, Sheffield, United Kingdom; ^5^Institute of Advanced Study, Technische Universität München, Munich, Germany

**Keywords:** dynamic contrast enhanced MRI, pharmacokinetic parameter inference, convolutional neural networks, ischaemic stroke, tracer kinetic modeling, contrast agent concentration, loss function

## Abstract

**Background and Purpose:** The T1-weighted dynamic contrast enhanced (DCE)-MRI is an imaging technique that provides a quantitative measure of pharmacokinetic (PK) parameters characterizing microvasculature of tissues. For the present study, we propose a new machine learning (ML) based approach to directly estimate the PK parameters from the acquired DCE-MRI image-time series that is both more robust and faster than conventional model fitting.

**Materials and Methods:** We specifically utilize deep convolutional neural networks (CNNs) to learn the mapping between the image-time series and corresponding PK parameters. DCE-MRI datasets acquired from 15 patients with clinically evident mild ischaemic stroke were used in the experiments. Training and testing were carried out based on leave-one-patient-out cross- validation. The parameter estimates obtained by the proposed CNN model were compared against the two tracer kinetic models: (1) Patlak model, (2) Extended Tofts model, where the estimation of model parameters is done via voxelwise linear and nonlinear least squares fitting respectively.

**Results:** The trained CNN model is able to yield PK parameters which can better discriminate different brain tissues, including stroke regions. The results also demonstrate that the model generalizes well to new cases even if a subject specific arterial input function (AIF) is not available for the new data.

**Conclusion:** A ML-based model can be used for direct inference of the PK parameters from DCE image series. This method may allow fast and robust parameter inference in population DCE studies. Parameter inference on a 3D volume-time series takes only a few seconds on a GPU machine, which is significantly faster compared to conventional non-linear least squares fitting.

## 1. Introduction

Dynamic contrast-enhanced magnetic resonance imaging (DCE-MRI) is an effective dynamic imaging technique that can be used to study microvascular structure *in vivo* by tracking the diffusion of a paramagnetic contrast agent such as gadopentate dimeglumine (Gd-DTPA) over time (1). By collecting a series of T_1_-weighted MR images at intervals of a few seconds, the uptake and washout of the administered contrast agent can be observed in the imaged tissue, resulting in characteristic intensity-time curves across different tissues (2). Vascular and cellular regularities in human body usually have a strong impact on the local vascular perfusion and permeability. To this end, DCE imaging has been used as a promising tool for clinical diagnostics of brain tumors, multiple sclerosis lesions, and several neurological disorders that lead to disruption and breakdown of blood-brain barrier (BBB) (3–6). In DCE-MRI, changes in contrast agent concentration are determined from changes in signal intensity over time, and then regressed through the use of tracer kinetic (TK) models to estimate pharmacokinetic (PK) parameters which characterizes the vascular permeability and tissue perfusion (7, 8).

**Figure 1:**
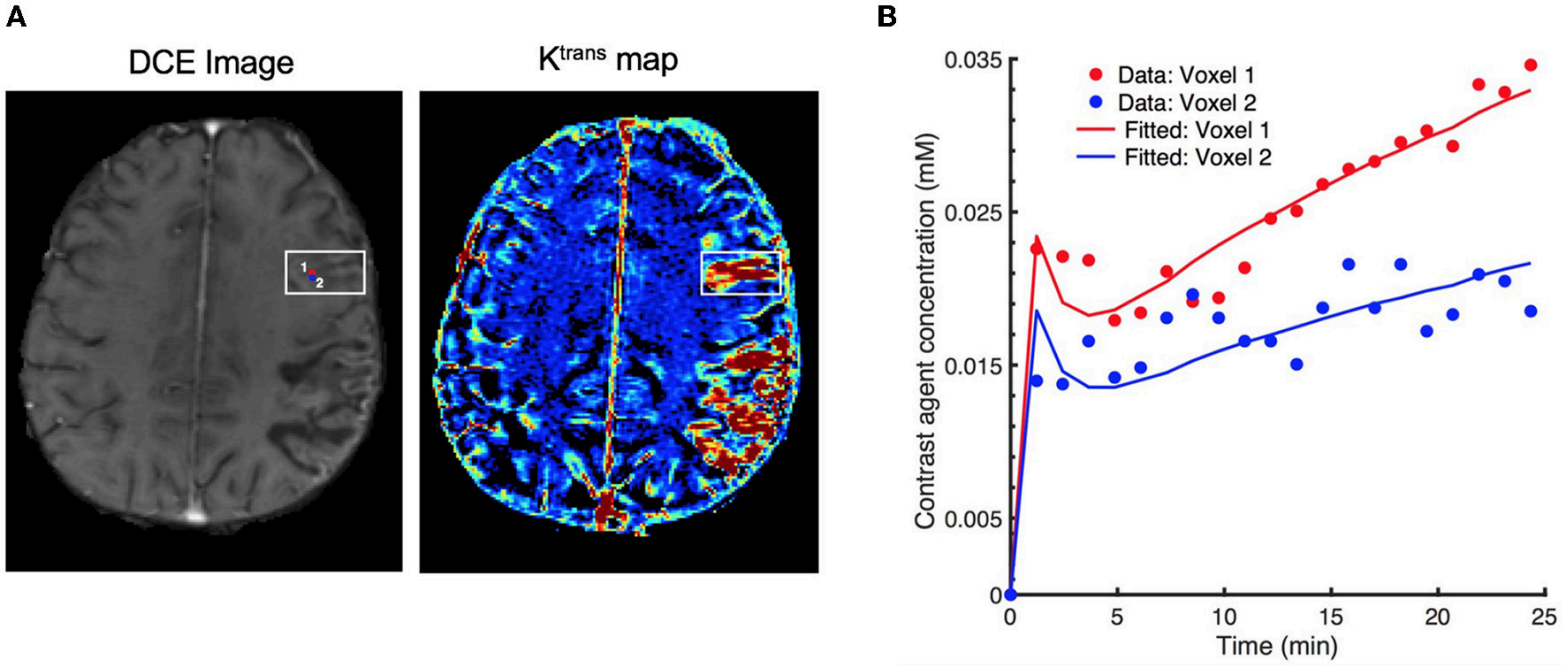
Effect of signal noise on the resulting parameters with conventional fitting models. **(A)** an examplary DCE image (left) displaying two neighboring voxels (marked by red and blue circles) in the stroke region, and the corresponding Ktrans maps (right), **(B)** resulting fitted contrast agent concentration curves for these two voxels using Extended Tofts model. Although the neighboring voxels are spatially very close to each other (only 1-pixel away), the observed concentration data are different due to the excessive signal noise. Eventually, there is a substantial difference in the fitted concentration curves and parameter values (*K*^trans^ = 6.18 × 10^−3^min^−1^ for voxel 1, and *K*^trans^ = 2.48 × 10^−3^min^−1^ for voxel 2).

One of the key limitations of TK modeling methods is that they are simply based on the fitting of voxelwise PK parameters to contrast agent concentration-time curves (9). The fitting is usually performed using a nonlinear least squares (NLS) approach. However, the acquired voxelwise concentration-time curves are generally very noisy and involve only a small number of sampling points, hence the model fitting may yield parameter estimates with large variance as well as considerable bias (see Figure 1 for an exemplary representation of this limitation). Moreover, an iterative NLS solver may converge to erroneous solutions since the NLS objective is not convex and can have multiple local minima (10). Another major drawback is that the voxelwise model fitting is computationally demanding considering the thousands of voxels in a single MR slice (11). More sophisticated approaches (10, 12) were also proposed based on Bayesian theory of statistical inference of the DCE parameters for the fitting of nonlinear models. Unlike the standard NLS regression, these approaches exploit the spatial information of the neighboring voxels and provide reduce variability of parameters in local homogeneous regions. However, the bottleneck is their drastically increased computation time, usually taking hours for the estimation of parameters on a single DCE scan.

Machine learning (ML) methods have been extensively used in the medical imaging community for several tasks (13) such as parameter estimation, disease classification, segmentation, so on. Recently, a random forest regression based method (14) was proposed to estimate accurate spectral parameters in MR spectroscopy. Deep learning methods (15–17) have recently gained large popularity and achieved predominantly state-of- the-art results in the medical imaging field including various image-to-image translation tasks (18–20). A deep neural network based approach for perfusion parameter estimation (21) was first proposed for dynamic susceptibility contrast (DSC) MRI without requiring a standard deconvolution process.

**Figure 2:**
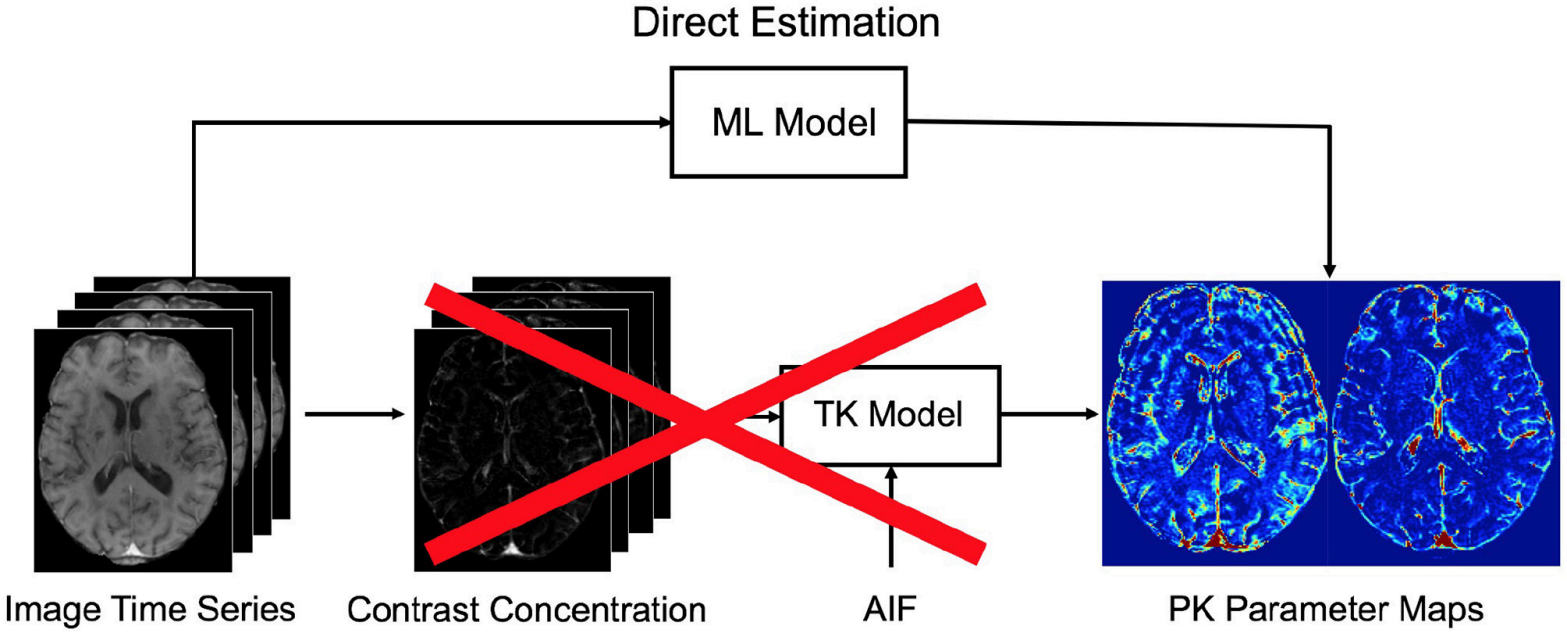
The conventional pipeline of pharmacokinetic parameters for DCE-MRI. Our proposed machine learning (ML) model allows to directly infer the parameters from the acquired DCE image time series. To this end, the intermediate computational steps—i.e., conversion to contrast concentration, extraction of AIF, and fitting to a tracer kinetic (TK) model—can be eliminated when applied on a test data. We note that in our approach a specific TK model can be still used to estimate target parameter values during training.

To alleviate the aforementioned limitations in DCE-MRI, we present a direct and fast PK parameter estimation method which introduces several concepts from machine learning. Our proposed approach can directly infer the PK parameters from the observed signal intensity over time. In order to achieve this, we first train a deep convolutional neural network (CNN) to learn the underlying mapping – or relation – between intensity image-time series and PK parameters using a large training data consisting of millions of voxels taken from the brain DCE dataset. In our method, the target PK parameters used in training step can be either estimated by any existing tracer kinetic models, or can be defined with reference values depending on a specific biomarker or disease that has been built on one specific type of model. Our method can intrinsically provide the following advantages over the conventional model fitting based parameter estimation approaches:

- The proposed method can directly estimate the corresponding physiological perfusion parameters when only observed signal intensities over time given, which eliminates several intermediate computation steps of the conventional pipeline as illustrated in Figure 2.
- Our method serves as a high-level parameter estimation model such that we can train a network from which we expect to yield parameter estimates as close as the target values that are obtained using any optimization approach, e.g., standard NLS fitting, regularized Bayesian estimation methods, etc.
- Due to its strong generalization ability, this method shows increased robustness to signal noise and outliers, and it can significantly mitigate the effect of irregularity and discontinuity problem which is quite apparent in the parameter maps estimated by conventional NLS fitting.
- The parameter estimates obtained by the proposed approach yields improved statistically significant differences between different tissue types, which can ultimately allow better discrimination of normal and pathological regions in stroke analysis.
- Compared to conventional fitting methods, the PK parameter inference with our ML based approach is computationally faster, taking only seconds on an entire 3D DCE-MRI volume.

## 2. Materials and Methods

### 2.1. Dataset and Preprocessing

#### 2.1.1. Patients

Fifteen patients were recruited for this study. The patient cohort presents first clinically evident mild (i.e., expected to be non-disabling) ischaemic stroke from the local stroke service. The patients were over 18 years old and had a definite diagnosis of ischaemic stroke. They were able to consent themselves, had an MRI scan at diagnosis and were medically stable enough to return for a DCE-MRI scan at between 1 and 3 months post-stroke and a follow-up after 1 year. All patients underwent clinical assessment by a stroke physician, diagnostic MR imaging and cognitive testing at presentation. An expert panel of stroke physicians and neuro-radiologists assessed each case in order to confirm the diagnosis of ischaemic stroke and classify the ischaemic stroke subtype. DCE-MRI was performed a minimum of 1 month after the stroke in order to avoid acute effects of the stroke on the local BBB (22). This study was approved by the Lothian Ethics of Medical Research Committee (REC 09/81101/54) and the NHS Lothian R + D Office (2009/W/NEU/14), and all patients gave written informed consent.

#### 2.1.2. MRI Acquisition

MR imaging was performed on a 1.5 T MRI scanner (Signa HDxt, General Electric (GE), Milwaukee, WI) using an 8-channel phased-array coil. Structural MR images for diagnostic purpose were acquired at first including axial T2-weighted (T2W; TR/TE = 6000/90 ms, FoV = 240 × 240 mm, acquisition matrix = 384 × 384, 1.5 averages, 28 × 5 mm slices, 1 mm slice gap), and axial fluid-attenuated inversion recovery (FLAIR; TR/TE/TI = 9000/153/2200 ms, FoV= 240 × 240 mm, acquisition matrix = 384 × 224, 28 × 5 mm slices, 1 mm slice gap).

DCE image series were acquired using a 3D T1W spoiled gradient echo sequence (TR/TE = 8.24/3.1 ms, flip angle = 12°, FoV = 240 × 240 mm, acquisition matrix = 256 × 192, slice thickness = 4 mm, 42 slices). Two pre-contrast acquisitions were carried out at flip angles of 2° and 12° to calculate pre-contrast longitudinal relaxation times (*T*_10_). An intravenous bolus injection of 0.1 mmol/kg of gadoterate meglumine (Gd-DOTA, Dotarem, Guerbet, France) was administered simultaneously with the start of 20 acquisitions with 12° flip angle and a temporal resolution of 73 seconds. The total acquisition time for DCE-MRI was approximately 24 minutes.

#### 2.1.3. Image Processing

**Figure 3:**
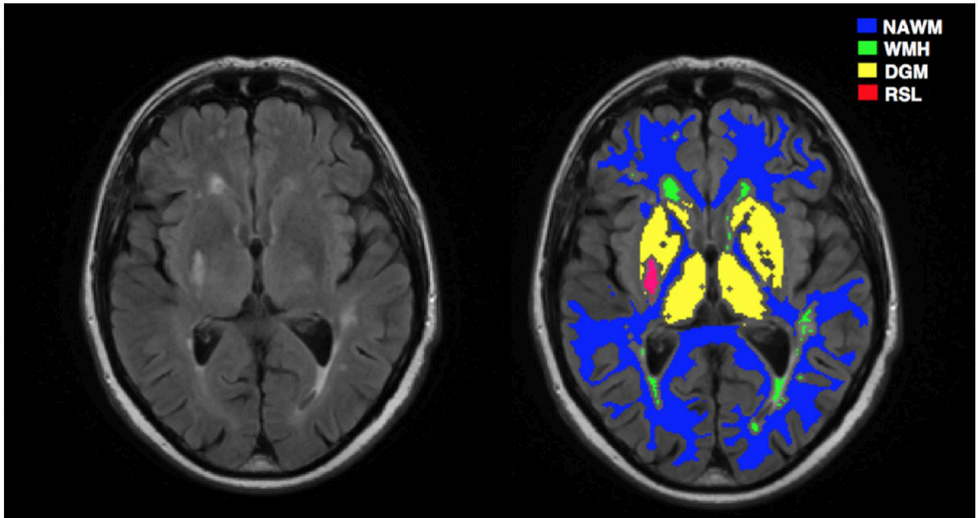
A representative MRI image and corresponding tissue segmentations. FLAIR image (left) and tissue masks superimposed on FLAIR image (right). NAWM, normal-appearing white matter; WMH, white matter hyperintensities; DGM, deep gray matter; RSL, recent stroke lesion.

For image preprocessing, we mainly followed the steps described in Heye et al. (22). First, all structural and DCE MR images were coregistered to the 12° pre-contrast image using rigid-body registration to correct for bulk patient movement. All small vessel features were determined according to agreed STRIVE standards (23). We employed a multispectral MRI data fusion and minimum variance quantization method (24) for the segmentation of white matter hyperintensities (WMH) and normal-appearing white matter (NAWM), and the resulting masks were manually refined. We used the “Region of Interest” tool of Analyze 11.0^TM^ (AnalyzeDirect, KS) to semi-automatically outline the old stroke lesions and recent stroke lesion (RSL) boundaries separately. Stroke lesion masks were checked for precision by a neuroradiologist; all other tissue masks were checked visually for accuracy and manually edited by an expert if necessary. Moreover, subcortical/deep gray matter (DGM) masks were generated automatically using a software pipeline as described in Heye et al. (22). In order to minimize any residual contamination of the DGM, the resulting mask was eroded by one voxel. Figure 3 depicts a representative FLAIR image and corresponding tissue segmentation.

### 2.2. DCE-MRI Analysis

Data collected at multiple flip angles were first used to calculate the *T*_10_ map based on the variable flip angle method proposed in Brookes et al. (25), given by

(1) $$\frac{1}{T_{\text{10}}}=\frac{1}{T_{\text{R}}}\text{ln}\left(\frac{S_{\text{R}}\text{sin}\alpha_{b}\text{cos}\alpha_{a}-\text{sin}\alpha_{a}\text{cos}\alpha_{b}}{S_{\text{R}}\text{sin}\alpha_{b}-\text{sin}\alpha_{a}}\right),$$

where *S*_R_ = *S*_*a*_/*S*_*b*_ with *S*_*a*_ and *S*_*b*_ denoting the signal intensities of the two pre-contrast acquisitions with flip angles $\alpha_{a}=2^{{}^{\circ}}$ and $\alpha_{b}=12^{{}^{\circ}}$, and *T*_R_ is the repetition time.

Dynamic DCE image series *S*(*t*) are converted to contrast agent concentration *C*_t_(*t*) through the steady-state spoiled gradient echo (SGPR) signal equation (26),

(2) $$S(t)=\frac{M_{\text{0}}\text{sin}\alpha_{b}\left(1-e^{-\left(K+L\right)}\right)}{1-\text{cos}\alpha_{b}e^{-\left(K+L\right)}}+\left(S(0)-\frac{M_{\text{0}}\text{sin}\alpha_{b}\left(1-e^{-K}\right)}{1-\text{cos}\alpha_{b}e^{-K}}\right),$$

where *K* = *T*_*R*_/*T*_10_, *L* = *r*_1_*C*_*t*_(*t*)*T*_*R*_, *r*_1_ is the contrast agent relaxivity taken as 4.2 s^−1^mM^−1^, *S*(0) is the baseline (pre-contrast) image intensity, and *T*_10_ and *M*_0_ are respectively the *T*_1_ relaxation and equilibrium longitudinal magnetization that are calculated from a pre-contrast *T*_1_ mapping acquisition.

For each subject, we extracted a vascular input function (VIF) from a region located on the superior sagittal sinus (SS) because partial volume effects and inflow artifact were reduced at this location compared to obtaining the arterial input function (AIF) from a feeding artery (22); the delay between arterial and venous responses is expected to be very small compared with the temporal resolution of our acquired data. Instead of selecting only a single voxel, we determined a 3 × 3 patch inside the SS region and estimated the VIF by averaging the time-signal intensities over the voxels within the patch. This enabled us to obtain more smooth variations in the DCE-MRI time course. We converted the whole-blood concentration *C*_b_(*t*) measured in the SS to plasma concentration using the formula *C*_p_(*t*) = *C*_b_(*t*)/(1−Hct) where Hct is the blood hematocrit measured in large arteries and assumed to be Hct = 0.45 as previously used in literature (22, 26, 27).

#### 2.2.1. Tracer Kinetic Models

Tracer kinetic modeling (28) is applied in DCE-MRI to provide a link between the contrast agent concentration and the physiological or so-called pharmacokinetic parameters, including the fractional plasma volume (*v*_p_), the fractional interstitial volume (*v*_e_), the volume transfer rate (*K*^trans^) at which contrast agent (CA) is delivered to the extravascular extracellular space (EES) from plasma space.

**Figure 4:**
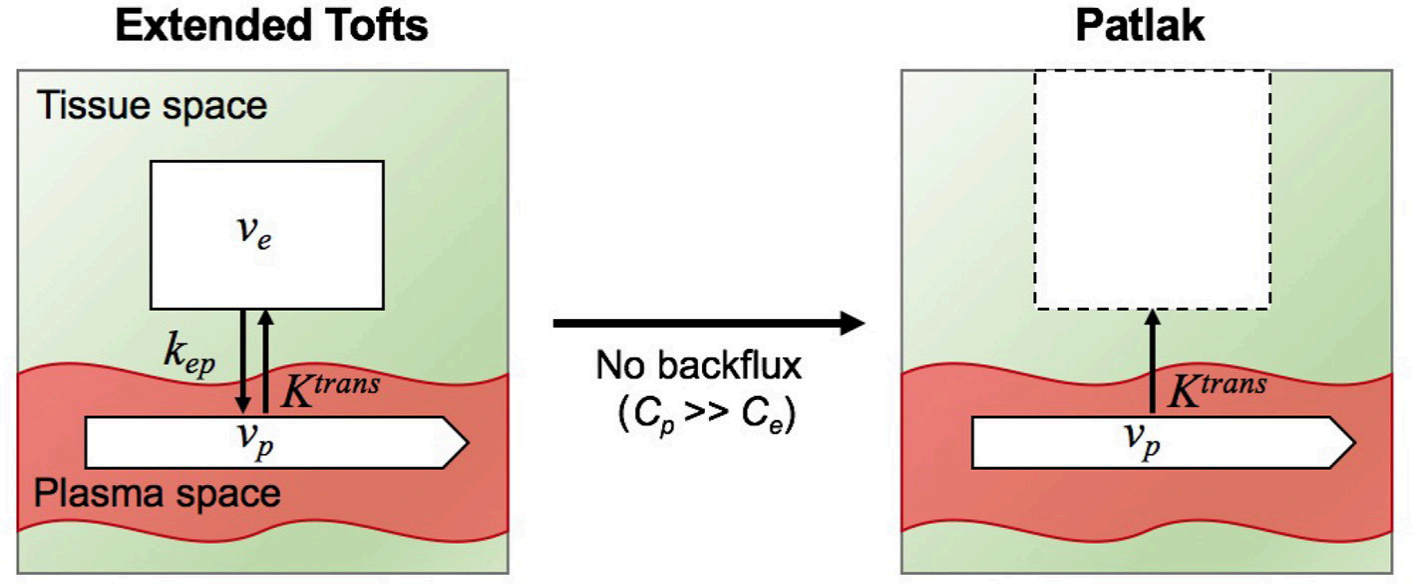
Illustration of two tracer kinetic models: Extended Tofts (left) and Patlak (right) model. Target parameters of DCE-MRI modeling are the contrast agent transfer rate from plasma space to tissue space *K*^trans^, the fractional plasma volume *v*_*p*_, the fractional interstitial volume *v*_*e*_, and transfer constant from the tissue space to the blood plasma *k*_ep_. Patlak model is related to Extended Tofts model through the assumption *C*_*p*_ >> *C*_*e*_ such that the backflux from the EES into the plasma space is negligible.

In this study, we fitted the following two models to the tissue concentration curves *C*_t_(*t*): (i) the extended Tofts model, (ii) the Patlak model. A schematic overview of the two models and their relationship is illustrated in Figure 4.

The extended Tofts (eTofts) model (29) mainly describes a highly perfused (*F*_p_ = ∞) two- compartment tissue model considering bidirectional transport between the blood plasma and EES. The concentration of contrast agent in the tissue is determined by,

(3) $$C_{\text{t}}(t)=v_{p}C_{\text{p}}(t)+K^{\text{trans}}\int_{0}^{t}C_{\text{p}}(\tau )e^{-k_{\text{ep}}(t-\tau )}d\tau ,$$

where $k_{\text{ep}}=K^{\text{trans}}/v_{e}$ represents the transfer constant from the EES back to the blood plasma. For the fitting of eTofts model, we used limited-memory Broyden-Fletcher Goldfarb-Shannon (l-BFGS) method for nonlinear minimization of the sum of squared residuals. The algorithm was run till convergence for a maximum of 30 iterations.

The Patlak Model (30) can be considered as a special case of the eTofts model, where the backflux from the EES into the blood plasma compartment is negligible. To this end, this model only allows measurement of the two parameters *K*^trans^ and *v*_*p*_ given by,

(4) $$C_{\text{t}}(t)=v_{p}C_{\text{p}}(t)+K^{\text{trans}}\int_{0}^{t}C_{\text{p}}(\tau )d\tau ,$$

An attractive feature of Patlak model is that the model equation in (4) is linear and model parameters can be fitted using linear least squares which has a closed-form solution, hence parameter estimation is fast (9).

### 2.3. Deep Learning for Pharmacokinetic Parameter Estimation

In this study, we consider the PK parameter inference in DCE-MRI as a mapping problem between intensity image-time series and parameter maps where the underlying mapping can be efficiently learned using deep CNNs. The proposed CNN aims at learning data-driven features with the use of convolutional feature filters to effectively detect the local spatio-temporal characteristics of the DCE time series. The extracted spatio-temporal features are desired to represent the underlying relation between the input and output of the network as much as possible.

Specifically, our CNN is trained to learn a mapping between *S*(*t*) and θ to output an estimate of PK maps $\tilde{\theta };\;\tilde{\theta }=f\left(S(t)|w\right)$, where **f** denotes the forward mapping of the CNN with the learned set of filter weights **w**. We note that set of parameters are represented by $\theta =\left\{K^{\text{trans}},v_{\text{p}}\right\}$ for Patlak model and $\theta =\left\{K^{\text{trans}},k_{\text{ep}},v_{\text{p}}\right\}$ for eTofts model.

#### 2.3.1. Loss Function

To learn the network weights (**w**) during training, we need to define an objective function (or loss function) to be minimized. In addition to the standard mean squared error (MSE) loss between the true PK parameter values θ and the estimated values $\tilde{\theta }$ which enforces high fidelity in parameter reconstruction, we simultaneously seek the fitted contrast agent concentrations of the PK parameters to be sufficiently close to the observed concentrations, *C*_t_(*t*). To this end, we formulate a new loss function which jointly incorporates these two loss criteria. Given a large number of training samples D of input-output pairs (*S*(*t*), θ), we train a CNN model that minimizes the following loss,

(5) $$ℒ(w)=\sum_{(S(t),\theta )\in D}\left(\|\theta -\tilde{\theta }{\|}_{2}^{2}+\|C_{\text{t}}(t)-f_{\text{tk}}(\tilde{\theta }){\|}_{2}^{2}\right),$$

where *f*_tk_ is the tracer kinetic model equation of either eTofts or Patlak model as formulated by Equation (3) or Equation (4), respectively.

#### 2.3.2. Network Architecture

**Figure 5:**
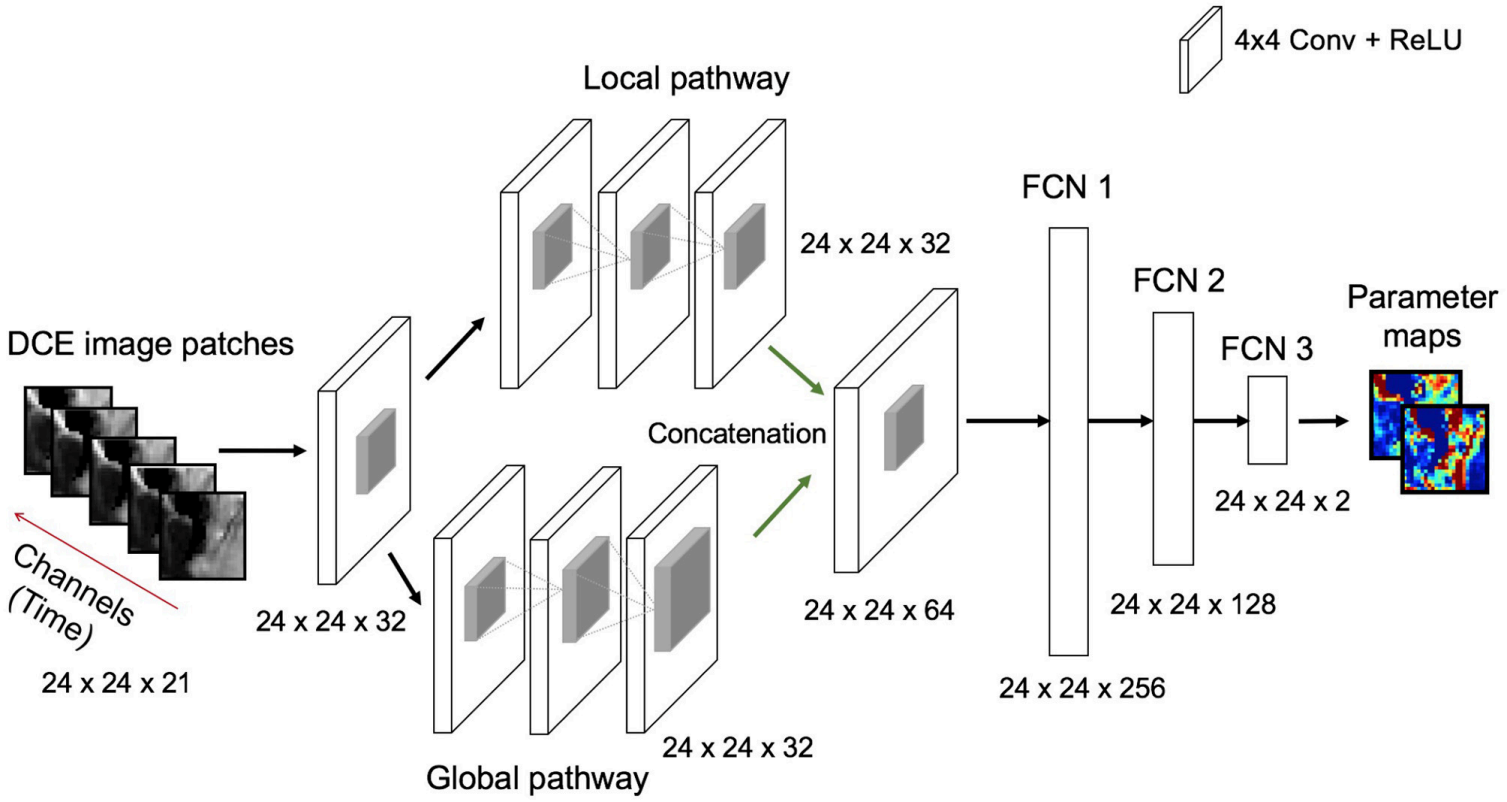
Illustration of the deep learning architecture used for the estimation of PK parameter maps given the DCE image patch-time series as input. All convolutional layers except concatenation layer learn 32 filters whereas concatenation layer learns 64 filters. Every convolutional layer involves a filter size of 4 × 4. ReLU is used as a non-linear activation function after each convolutional and fully connected layer. The size of the outputs from each layer operation [e.g., input, convolution and full connection (FCN)] are also displayed at the bottom of each layer.

We illustrate the network structure used in this study in Figure 5. The network takes DCE image-time series as input with a patch size of 24 × 24 × 21, where time frames are stacked as input channels. The first convolutional layer applies 2D filters to each channel individually to extract low-level temporal features which are aggregated over frames via learned filter weights to produce a single output per voxel. Inspired by the work on brain segmentation (31) and denoising in arterial spin labeling (32), our network consists of parallel dual pathways to efficiently capture multi-scale information after the first layer. The local pathway focuses on extracting details from the local image regions while the global pathway is designed to incorporate more contextual global information. The global pathway consists of 3 dilated convolutional layers with dilation factors of 2, 4, and 8, indicating increased receptive field sizes. Zero-padding is applied before every convolution operation to keep the spatial dimensions of the output equal to the input. The filter size of each convolutional layer including dilated convolutions is chosen as 4 × 4. The rectified linear units (ReLU) activation function (*f*(*x*) = max(0, *x*)) is applied after each convolution to introduce non-linearity into the mapping. Local and global pathways are then concatenated to form a multi-scale feature set. Following this, two fully-connected layers of 256 and 128 hidden nodes are used to determine the best possible feature combination that can accurately map the input to output of the network. Finally, the last fully-connected layer outputs the parameter estimates of a patch size 24 × 24 × *n*, where *n* is the number of kinetic model parameters. We emphasize that as our proposed network was structured to estimate outputs for every single voxel of the input patch, it is essential to keep the spatial dimensions of the input and output same throughout the network. Therefore, in our network we can consider a fully-connected (FCN) layer as a convolutional (CONV) layer with 1 × 1 convolutions.

#### 2.3.3. Network Training

Among all the follow-up scans we only selected one DCE-MRI scan per subject in our experiments. All these scans were acquired at between 1-3 months post-stroke. For each patients data, we neglected the first and last 5 image slices due to insufficient brain coverage. Among the remaining slices of each patient we randomly selected 20 slices to be considered in analysis. We note that these are the central 20 slices that contain most of the brain regions in overall. Following to this, each 2D DCE image slice was divided into overlapping patches of size 24 × 24 voxels with step size of 6 voxels. This resulted in a collection of approximately 12, 000 patches for every patients data. We applied the same procedure on contrast agent concentration data and target parameter maps required for network training.

**Figure 6:**
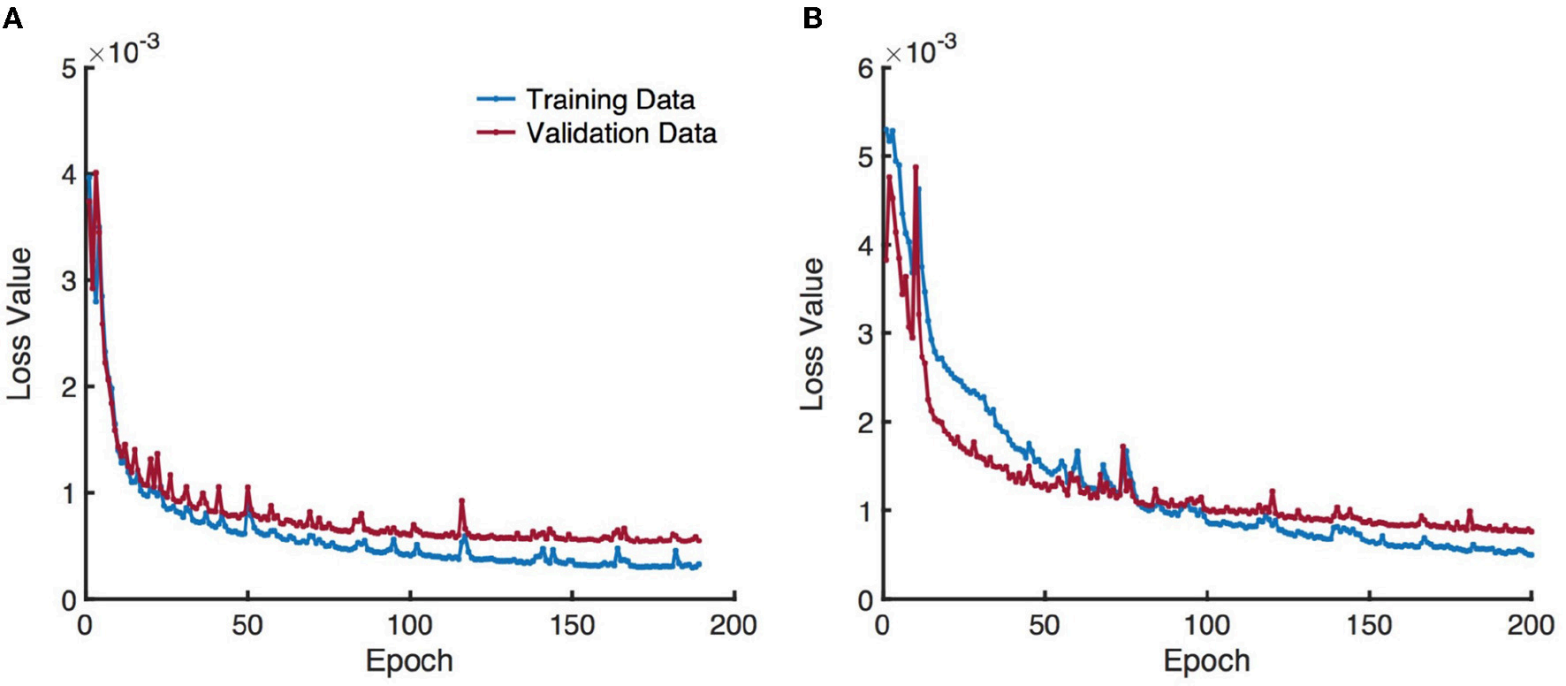
Training and validation loss over epochs obtained by training a CNN model using **(A)** Patlak and **(B)** eTofts model parameters. Gradual decrease in the loss indicates the efficiency of the network for learning useful representations related to the underlying mapping between the input and output.

All experiments were performed in a leave-one-subject-out fashion, i.e., 30 different networks were trained in total based on both Patlak and eTofts model parameters. Randomly chosen 10, 000 overlapping patches of each subject were split into training (80%) and validation (20%) sets. The networks were trained using the Adam optimizer with a learning rate of 10^−3^ and a decay rate of 10^−4^ for maximum number of 200 epochs and a mini-batch size of 1000 patches. Early stopping was applied to prevent poor generalization performance when the validation loss stopped improving within consecutive 15 epochs. In Figure 6 we provide two exemplary plots depicting the changes in training and validation loss over epochs for CNN trained on Patlak and eTofts models. Both losses show a decreasing trend and converge to a minimum. We implemented our code using Keras library with TensorFlow (33) backend, and experiments were run on a NVIDIA GeForce Titan Xp GPU with 12 GB RAM.

#### 2.3.4. Testing

Once the network is trained and network parameters are learned, DCE image-time series data of a test subject can be fed into the network to directly predict the PK parameters. Since the predictions are processed in a patch-wise manner, all overlapping 16 predictions of a neighborhood are averaged to obtain a final value for every individual voxel.

## 3. Results

### 3.1. Comparison of Pharmacokinetic Maps

**Figure 7:**
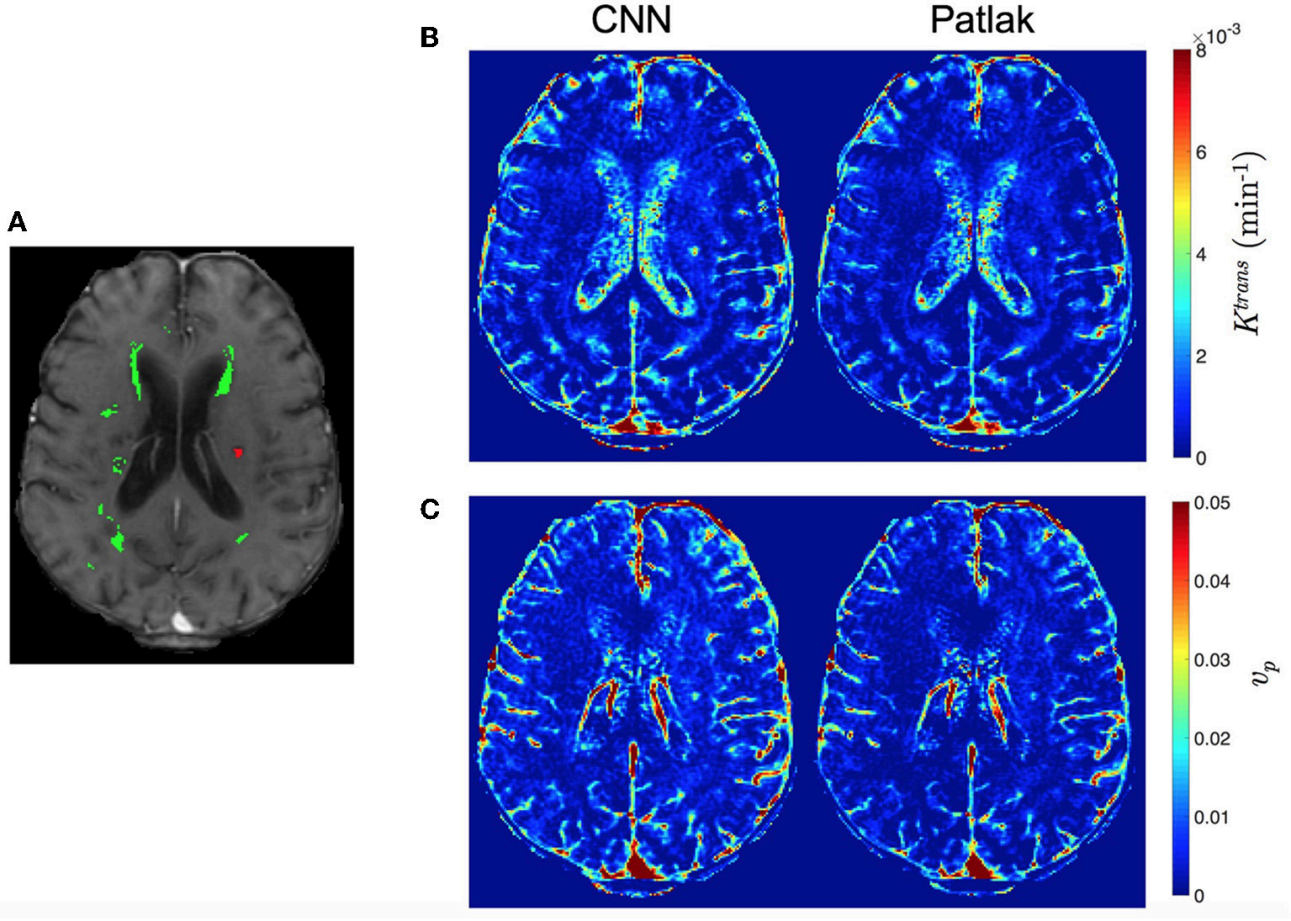
Comparison of qualitative PK parameter maps from a slice of a stroke patient data. **(A)** a DCE image slice on which the tissue masks are superimposed (WMH: green, RSL: red), **(B)** *K*^trans^ and **(C)** *v*_*p*_ parameter maps obtained by CNN model and Patlak fitting.

We compare the qualitative PK parameter maps obtained by Patlak model fitting, eTofts model fitting and CNN model trained by either Patlak or eTofts model. Figures 7B,C shows PK parameter maps of an exemplary slice of a patients data. In overall, the parameter maps by CNN model looks very similar with the Patlak model fitting. However, the CNN model produces higher estimates of *K*^trans^ in especially small RSL region as marked on the DCE image in Figure 7A. Moreover, the RSL region is more distinctive and can be discriminated well with respect to other tissues in both the parameter maps of CNN model. For numerical evaluation of output parameter maps, we used two evaluation metrics calculated within the entire brain region: Structural similarity index (SSIM) and normalized root mean square error (nRMSE). These values were calculated by considering the output maps of Patlak model as reference, shown in Figures 7B,C. For *K*^trans^, we obtain a high SSIM of 0.991 and a low nRMSE of 0.0144. For *v*_*p*_, SSIM is calculated as 0.973 and nRMSE is 0.0168.

**Figure 8:**
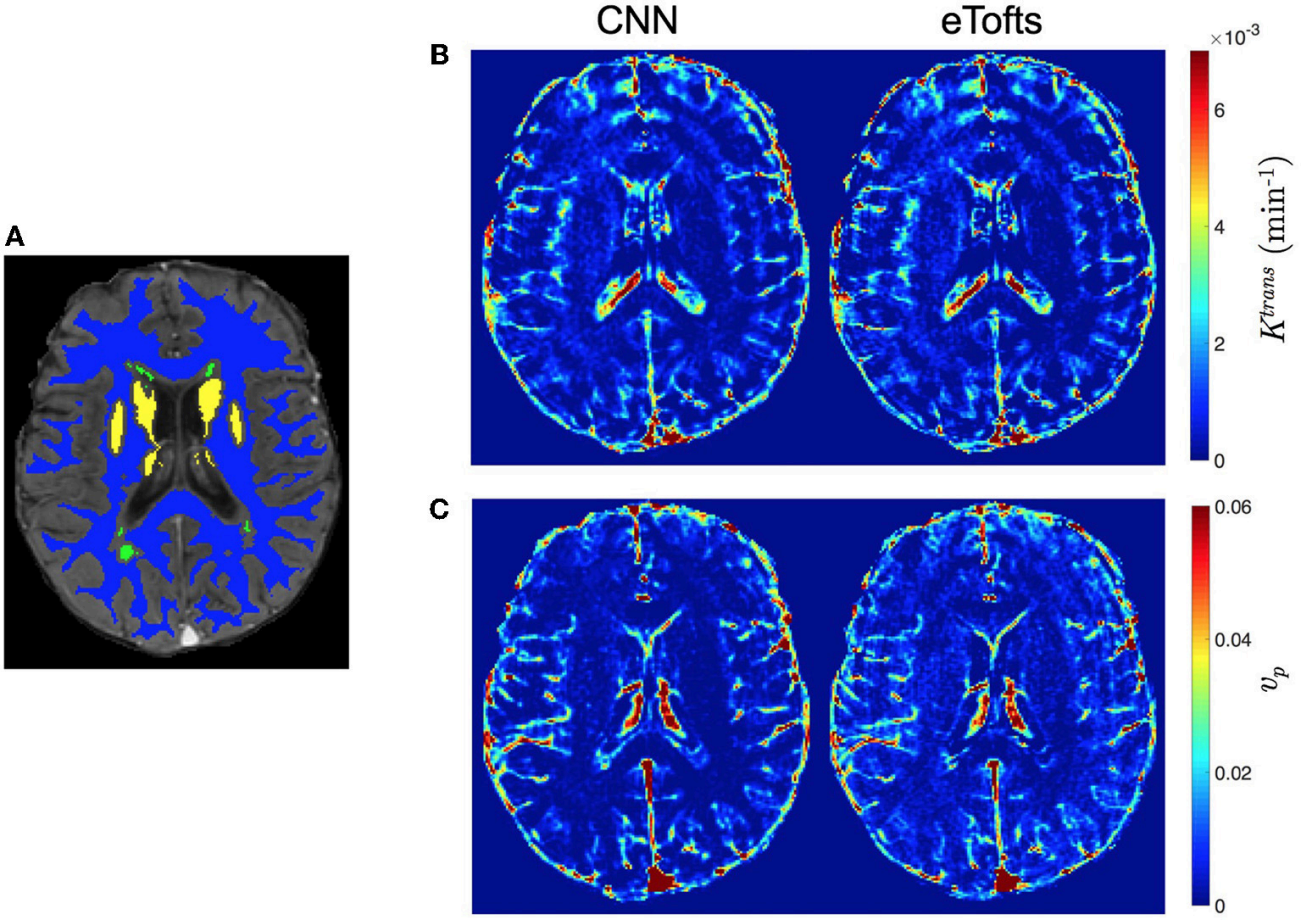
Comparison of qualitative PK parameter maps from a slice of a patient data with white matter hyperintensities. **(A)** a DCE image slice on which the tissue masks are superimposed (NAWM: blue, WMH: green, DGM: yellow), **(B)** *K*^trans^ and **(C)** *v*_*p*_ parameter maps obtained by CNN model and eTofts fitting. We remark that WMH represents the WM tissue associated with increased risk of dementia and cognitive decline.

Figures 8B,C demonstrates PK parameter maps of an exemplary slice of an another patients data fitted by eTofts model. The parameter estimates significantly match each other (for CNN and eTofts) in many of the tissue regions except NAWM as depicted on the DCE image in Figure 8A. As shown in Figure 8C, CNN model yields lower *v*_*p*_ values in comparison to eTofts model in NAWM. Hence, the discrimination of the NAWM with respect to WMH is more prominent. Quantitatively, when compared against the parameter maps obtained by eTofts model, CNN maps yield a SSIM score of 0.998 and 0.961 for *K*^trans^ and *v*_*p*_, respectively, while nRMSE is 0.0073 and 0.0156.

### 3.2. Fitting to the Observed Concentration -Time Series

We evaluate the accuracy of the fitting to the observed concentration-time series data. The fitted contrast agent concentration-time series were estimated via (3) and (4) by using the parameter estimates of Patlak, eTofts, and CNN models separately.

Table 1 demonstrates the quantitative comparison of the fitting to the observed contrast agent concentration time series data for different models in terms of nRMSE and SSIM. The metric values were calculated for every 2D slice of a subject's volume, and statistical values (mean ± std) were obtained using all 15 subject's data. The results indicate that standard Patlak and eTofts model can fit the data better compared to the CNN model trained with these models separately. However, the difference is not substantial that CNN model still achieves high accuracy with less than an average %2 fitting error.

**Table 1:** nRMSE (%) and SSIM statistics (mean ± std) obtained from concentration-time series data fitting. The SSIM value can vary between –1 and 1, where 1 indicates perfect similarity.

	**Models**			
**Metric**	**Patlak**	**eTofts**	**CNN: Patlak trained**	**CNN: eTofts trained**
nRMSE (%)	1.1200 ± 0.5225	1.0575 ± 0.5744	1.6398 ± 0.6878	1.7360 ± 0.7408
SSIM	0.9812 ± 0.0141	0.9835 ± 0.0127	0.9750 ± 0.0138	0.9719 ± 0.0162

**Figure 9:**
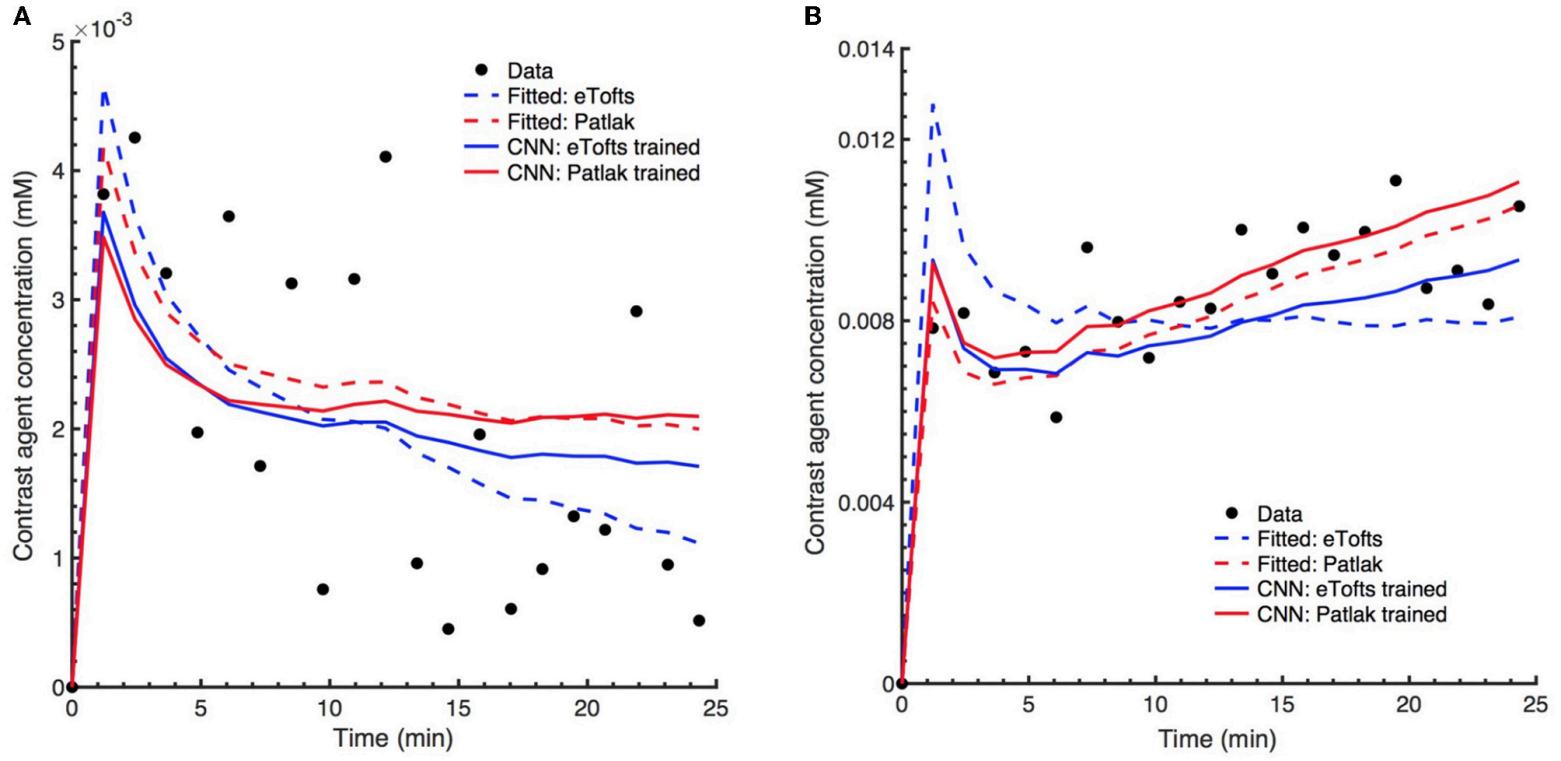
Comparison of model fits to the observed patient data. Exemplary concentration-time curves for tissue regions **(A)** NAWM and **(B)** RSL. In general, the CNN model trained by either Patlak or eTofts model parameters can fit the data similarly well when compared with fitted Patlak model. More interestingly, the CNN model trained by eTofts is in better alignment with observed data while the fitted eTofts is not sufficient to describe the data.

Figures 9A,B shows the fitting of contrast concentration (in mM) for the NAWM and RSL regions in a single patient data. In general, the CNN model trained by either Patlak or eTofts model parameters can fit the data similarly well when compared with Patlak and eTofts model. An interesting observation in Figure 9B is that the eTofts model does not fit the observed data well whereas the fitting obtained by CNN model trained on eTofts parameters is more accurate.

### 3.3. Statistical Analysis of PK Parameter Estimation

**Figure 10:**
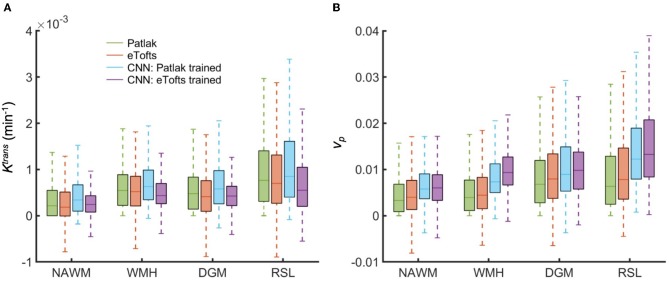
Comparison of fitted and estimated PK parameters between tissue types obtained from all subjects data. Box plots shows the distribution of **(A)** *K*^trans^ and **(B)** *v*_*p*_ in NAWM, WMH, DGM, and RSL. Box plots depict the median with a colored horizontal line for every method in comparison. Remarkably, CNN model trained on Patlak model results in *K*^trans^ and *v*_*p*_ values which show statistically significant differences between tissue types.

We perform statistical analysis of the parameter estimates on different tissues. A comparison between tissue types is shown in Figure 10. We assessed the statistical significance of the differences using the paired Wilcoxon signed rank test. For Patlak and eTofts model, all differences between tissue types were significant (*p* < 0.001) except for *K*^trans^ in DGM and WMH, and *v*_*p*_ in WMH and RSL. For CNN model trained on Patlak model parameters, all differences of *K*^trans^ between tissue types were significant including the difference between WMH and DGM (*p* = 3.4 × 10^−4^). The difference between WMH and RSL for *v*_*p*_ is again statistically significant with *p* = 1.6 × 10^−5^. The CNN model trained on Patlak generally tends to overestimate the *K*^trans^ and *v*_*p*_ parameters compared to either Patlak or eTofts model. The difference between them are significant with *p* < 0.001, and this is valid for all tissue types except DGM (*p* = 0.021 for *K*^trans^). On the other hand, the CNN model trained with eTofts parameters yield underestimated *K*^trans^ and overestimated *v*_*p*_ values when compared against either Patlak or eTofts model. The underestimation of *K*^trans^ by CNN is statistically significant for all tissue types except WMH (*p* = 0.317). The overestimation of *v*_*p*_ by CNN is significant for all tissue types (*p* < 0.001).

**Figure 11:**
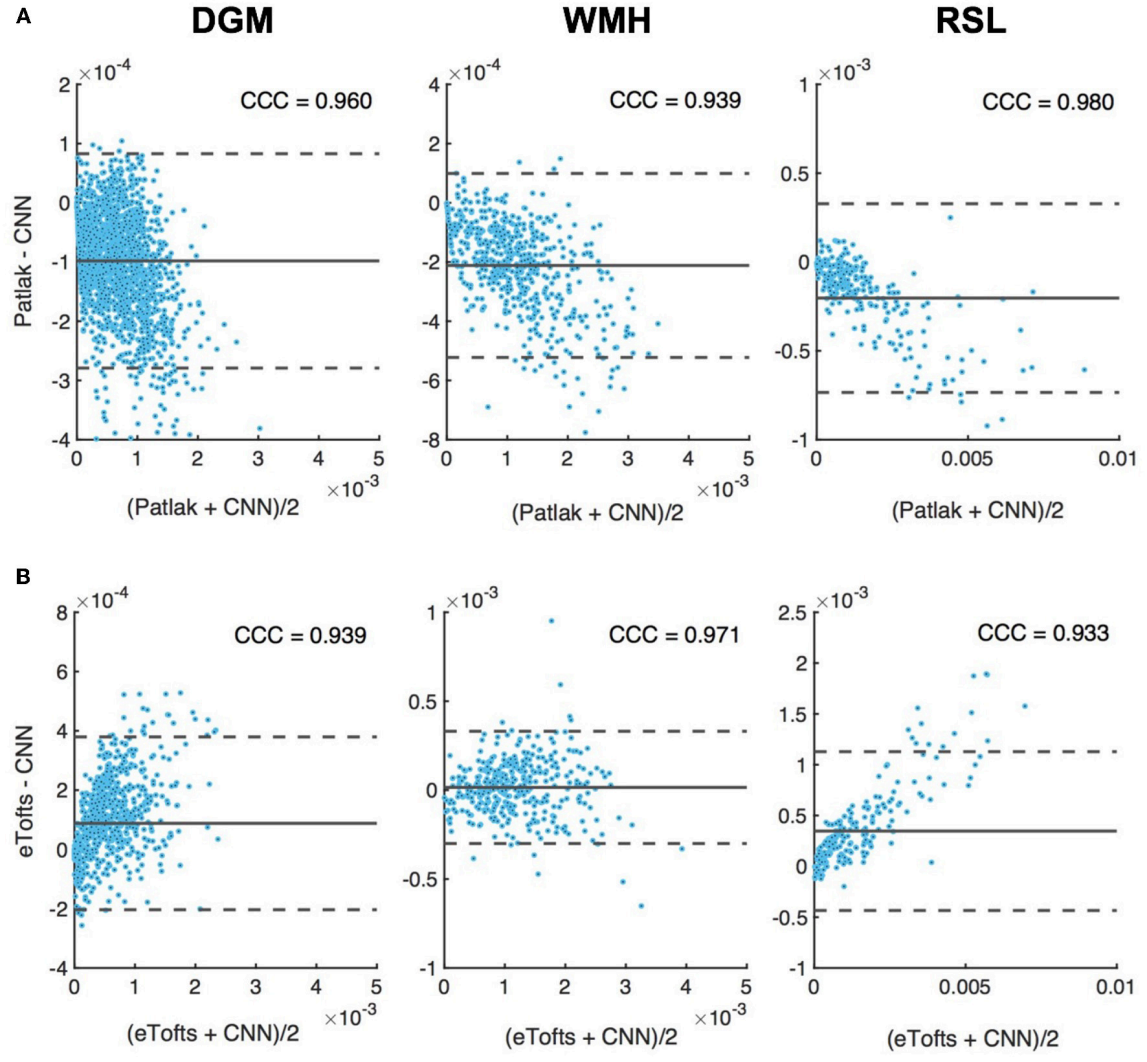
Bland-Altman plots of permeability *K*^trans^ parameters in different tissues, DGM (left), WMH (middle), RSL (right). Difference (y-axis) in *K*^trans^ values between CNN model and **(A)** Patlak, and, **(B)** eTofts fitting is plotted against the mean values of the two (*x-axis*). Solid gray line indicates mean difference (*mdiff*). Top and bottom gray dashed lines correspond to upper and lower margins of 95% limits of agreement estimated by *mdiff* ±1.96 × SD, SD = standard deviation. Units for horizontal and vertical axes are in min^−1^. The computed Lin's concordance correlation coefficient (CCC) values are displayed at top-right corner of each plot.

Figure 11 depicts the Bland-Altman plots of *K*^trans^ values in three different tissues (DGM, WMH, RSL) obtained from a patient's data. As can be observed in Figure 11A, when compared against the Patlak model, CNN model trained with Patlak tends to slightly underestimate the *K*^trans^ in DGM and overestimate the values in WMH and RSL. Figure 11B indicates that *K*^trans^ are underestimated by CNN trained with eTofts in DGM and RSL. The values in WMH highly match with Patlak fitting showing no systematic difference. In general, the results in Bland-Altman plots agree with the statistical results as shown in Figure 10, meaning that systematic differences are observable between the estimates of CNN and model fitting although concordance correlation coefficients (CCCs) indicate a strong agreement.

## 4. Discussion

The results of this study show that a CNN based ML model can yield PK parameter estimates that are comparable to traditional model fitting. As depicted in Figures 7, 8, the qualitative parameter maps estimated by CNN models match highly with the ones obtained by conventional TK model fitting methods. Moreover, ML based models can enable better discrimination of different brain tissues. As can be seen in Figure 7, small stroke lesion is more visible with higher *K*^trans^ values assigned to this region. In addition to this, the discontinuities of parameter values arising especially at highly perfused regions (i.e., vessels) can be mitigated by CNN model, and more smoother local areas are produced in these regions as shown in Figures 7, 8.

Statistical analysis in Figure 10 indicate that significant differences between tissue types can be achieved by CNN model whereas both Patlak and eTofts model fail in quantitatively differentiating some of the tissues pairwise including WMH-DGM. Especially higher *K*^trans^ values are generally assigned to stroke regions i.e., RSL, allowing better discrimination of these areas against non-stroke regions. To this end, the proposed ML model can be an appropriate parameter inference model for quantification of subtle BBB disruption where measuring low-level BBB permeability is vital in several diseases, including cerebral small vessel disease, lacunar stroke and vascular dementia. Another interesting observation is that the plasma volume *v*_*p*_ values estimated by CNN model in WMH are considerably greater than in normal-appearing WM areas. This may result in improved identification of the hyperintensity areas from the surrounding normal appearing WM tissue. WM hyperintensities are usually regarded as surrogates of small vessel disease and frequently seen in elderly people (34).

The major advantage of ML based model is that the parameter inference of a voxel belonging to a specific tissue type is performed by taking into account many other training samples, or voxels, of the same tissue type. Therefore, if the signal time series of a target voxel is subject to high noise, it is likely that a parameter value associated with the voxels that show similar signal trends and located in the same tissue type can be assigned to the target voxel. One example relevant to this observation can be seen in Figure 9B, where the fitted concentration time curves are provided for a ROI inside the RSL region of a patients data. Here, the eTofts model does not provide a good fit to the measured signal and the fitted concentration-time curve describes more a vascular region (i.e., blood vessel). However, the fit of the CNN model trained with eTofts model parameters can produce significantly better fit to the observed data, and the fit resembles more an RSL region, which is highly similar with the fits by Patlak and CNN model trained by Patlak model parameters. These findings reveal better generalization ability of ML models (35) which can extract and learn important tissue specific features from a large cohort of training examples. However, it should be noted that the correction of misfit of concentration time curves in Figure 9B does not point out an unique feature of our CNN based approach, but rather shows a specific case. The avoidance of a misfit with the CNN network primarily depends upon the model and optimization approach on which the network is trained.

Another observation from Figure 11 also signifies the tendency of CNN model to produce parameter estimates close to a mean value of parameter distribution learned from many training voxels within in a specific tissue. Here, when compared to the standard Patlak model parameters, we observe overestimated values in especially WMH and RSL region where the *K*^trans^ usually has higher values. The overestimation in some of the voxels within these tissues is presumably caused by the relatively lower parameter values estimated by Patlak model due to significant signal noise and fitting to the local minima. In this regard, systematic differences between CNN model estimates and standard NLS fitting are inevitable because the parameter estimates by NLS fitting is not optimal and usually produces a parameter distribution from a high range of values within the voxels of a specific tissue, as it can be seen in Figure 10. We anticipate that more accurate evaluation of systematic differences can be obtained using the synthetic DCE dataset where the ground truth parameters are known.

As mentioned before, one of the key advantages of our method is its utility to avoid intermediate computation steps of parameter inference in DCE-MRI by replacing it with a direct inference model. Although we use two existing TK models to estimate the reference parameters, based on the specific DCE application, one can use different TK models in literature (9) to infer the PK parameters to be used during training of the CNN network. If available, the network can be also trained using ground truth parameter values. In addition to this, as previously done in Banerji et al. (36) and Bosca and Jackson (37), synthetic DCE phantom data can be generated by simulating the signal equation and TK model equations with the PK parameters estimated from real patient's data, and a CNN model can be trained based on the synthetic data and corresponding parameter maps. With this approach, more realistic synthetic DCE datasets can be generated by taking into account the acquisition noise and motion artifacts. The generated synthetic datasets may be utilized to train a network which can be later tested on *in vivo* DCE dataset to obtain less noise-sensitive parameter estimates.

**Figure 12:**
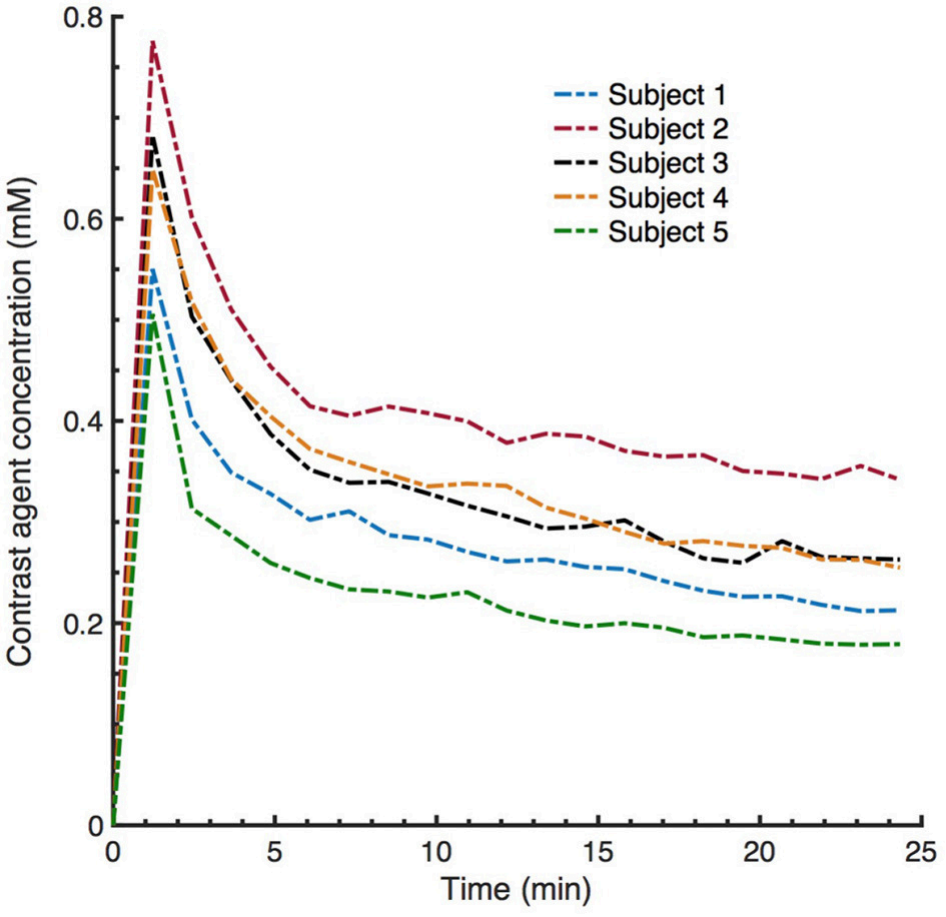
Examplary subject specific vascular input functions (VIFs) extracted from averaging a few voxels located on the superior sagittal sinus (SS). Although the VIFs appear to have similar shapes, the peaks and steady state signal can have varying magnitude (contrast agent concentration).

In conventional DCE-MRI analysis pipeline, subject-specific AIF extraction from a ROI of a feeding artery is one of the essential steps for the estimation of kinetic parameters (28, 38). In this study, we demonstrate that CNN based ML model can estimate PK parameters by no need of subject-specific AIF of the test subject without introducing any significant bias in the parameter estimation. Although this can be seen as one of the benefits of our model, we should remark that the data used in this work is a part of a population study where the temporal resolution and other parameters related to DCE acquisition and contrast injection are fixed in all subjects. However, as can be clearly seen in Figure 12, the subject-specific AIFs of our dataset usually have varying magnitudes of the peak and steady-state signal even though the time point where the signal reaches the peak is similar for all subjects. The signal pattern of the AIF curves are directly related to signal time intensities through Equation (2), hence the trained network can intrinsically learn the relation between the AIF and target parameters via the mapping between the input and output of the network and the designed loss function which takes into account the underlying TK model through its equation. On the other hand, the performance of proposed model on a mixed data—ideally involving DCE image series acquired with different acquisition parameters and protocols—can be subject to further investigation. For parameter estimation with a model trained on a mixed data, we anticipate that a bi-CNN input model similar to as proposed for DSC-MRI (21) might be a good approach to avoid bias and error in parameter estimation. In that setting, the DCE image time series and other acquisition parameters—including AIF—of both training and test subjects can be given to the network as two separate inputs.

We emphasize that our CNN model is not trained on a entire brain basis, but on individual time series. Out of the 15 patient datasets we extract more than 160 million training samples, i.e., number of total voxels in the training dataset. Moreover, our network architecture is not very deep and we demonstrate that this huge number of training samples is sufficient to train a network that generalizes well, where the inability to generate reproducible results is not an issue. Nevertheless, a wider sampling of pathological cases and MRI artifacts in training data is highly desirable and is one of the major direction for improvements of the proposed approach. The proposed model can—even should—be updated accordingly when applied to a larger pool of patient datasets. In general, based on the literature in ML, we anticipate that CNN-based ML models perform better when there is a high correlation and similarity between the training and test data. The dataset used in this study for both training and testing involve voxels from different type of tissues, e.g., healthy and pathological tissues, containing a good mixture of different tissue characteristics. There is a high similarity between the temporal profiles of training and test image patches, hence, the performance of CNN is very stable and robust. However, a poor generalization issue may usually arise in a scenario that the training data only consists of healthy tissue voxels whereas the unseen test data with pathological tissues is tested using the trained model. In this scenario, since the model is not trained with sufficient number of pathological samples, it is quite likely that the CNN model shows a poor performance on these test data comprised of non-healthy tissues. In principle, in order to obtain a stable CNN model, it is necessary to constitute a training data pool according to the demands or expectation from such a prediction model in our specific clinical applications. For instance, if we aim to discriminate well the acute/post-acute stroke regions, our training data should contain high number of voxels from both stroke and non-stroke regions.

Nevertheless, we should discuss the several limitations of this study. First, although ML based methods can have strong generalization ability, the bias is also inevitable when tested on an unseen data because the model is always trained using other subject's data without any access to test data. Second, the performance of our method may be improved depending on the input patch size and filter size of the network. Moreover, we only considered 2D convolution operations, however, 3D convolutions may produce better results when more spatial context information are extracted. Third, further investigation on synthetic data is required to perform accurate assessment of error and bias when the ground truth parameter values are known. Lastly, our current approach is sensitive to variation in acquisition parameters, especially temporal resolution, i.e., number of time points in DCE data. One feasible solution to the variations in temporal resolution across multiple datasets is to apply interpolation on time. In practice, we may interpolate all training data acquired with various temporal resolutions to a common temporal resolution so that a test data with completely different temporal resolution can be also fed into the trained network to produce parameter estimates.

In conclusion, this study shows that a ML based direct inference approach can estimate PK parameters that are comparable to the conventional model fitting in DCE-MRI. Our results, based on a sample of mild ischaemic stroke patients, demonstrate the efficiency of CNN model to enable better discrimination of brain tissue types. Specifically, our proposed ML based method has the potential to improve the current quantitative analysis of DCE-MRI studies due to its increased robustness to noise. Significant difference of permeability parameters between stroke and non-stroke regions may ultimately effect the stroke medical decision process. Finally, parameter inference of the proposed model on a 3D brain volume is considerably faster than the standard NLS fitting, demonstrating the applicability of such models in clinical practice. Considering such faster computation time, clinical experts may perform parameter inference using various TK models in parallel to benefit from making more detailed analysis between different models.

## Author Contributions

CU study concept, data analysis, experimental design, writing of manuscript. DD experimental design, writing of manuscript. MT study concept, experimental design. MV image preprocessing. PA and SM data collection. JW funding. BM study concept, writing of manuscript, funding. All authors contributed to reviewing and editing the final manuscript.

### Conflict of Interest Statement

DD is affiliated to GE Global Research as a doctoral student. The remaining authors declare that the research was conducted in the absence of any commercial or financial relationships that could be construed as a potential conflict of interest.
